# MLVA typing of *Mycoplasma hyopneumoniae* bacterins and field strains

**DOI:** 10.1136/vetreco-2015-000117

**Published:** 2015-10-09

**Authors:** P. Tamiozzo, R. Zamora, P. M. A. Lucchesi, A. Estanguet, J. Parada, A. Carranza, P. Camacho, A. Ambrogi

**Affiliations:** 1 Departamento Patología Animal, Facultad de Agronomía y Veterinaria, Universidad Nacional de Río Cuarto, Ruta 36 km 601. Río Cuarto, Córdoba, C.P. 5800, República Argentina; 2 Consejo Nacional de Investigaciones Científicas y Técnicas (CONICET), Buenos Aires, República Argentina; 3 Laboratorio de Inmunoquímica y Biotecnología, Centro de Investigación Veterinaria de Tandil, Facultad de Ciencias Veterinarias, Universidad Nacional del Centro de la Provincia de Buenos Aires, Tandil, Buenos Aires. Paraje Arroyo Seco s/n, C.P. 7000, República Argentina

**Keywords:** Swine, Enzootic pneumonia, Mycoplasma hyopneumoniae, genetic subtypes, MLVA

## Abstract

Because of the lack of information about both the genetic characteristics of *Mycoplasma hyopneumoniae* commercial vaccines and their relationship with field strains, the authors attempted to identify genetic subtypes of some *M hyopneumoniae* bacterins, and to compare them with *M. hyopneumoniae* field strains. Six commercial *M hyopneumoniae* bacterins and 28 bronchoalveolar lavages from pigs at slaughter from three herds were analysed by Multiple-Locus Variable number tandem repeat Analysis (MLVA) on *p146R1*, *p146R3*, *H4*, *H5* and *p95* loci. The results obtained showed the presence of more than one *M hyopneumoniae* genotype in some pigs and also in one of the bacterins analysed. It is also worth noting that MLVA typing allowed the distinction among circulating field strains and also when comparing them with vaccine strains, which, knowing the relatedness among them, could be useful in the research of the efficacy of the vaccines.

## Introduction

*Mycoplasma hyopneumoniae* is the primary agent involved in porcine enzootic pneumonia. Infections with *M hyopneumoniae* are highly prevalent in almost all swine-producing areas, causing significant economic losses to the pig industry worldwide (Thacker 2006). Control of *M hyopneumoniae* infections can be accomplished in several ways, mainly by optimisation of management practices and the use of antimicrobials and vaccines (Maes and others 2008). However, the protection induced with current *M hyopneumoniae* bacterins is incomplete because these vaccines do not prevent colonisation (Haesebrouck and others 2004).

On the one hand, a wide range of *M hyopneumoniae* vaccines are currently commercially available, and most of them, if not all, are bacterins. Although previous studies have evaluated pig immunisation using either homologous and heterologous vaccines (Villarreal and others 2012) or protective efficacy against high-virulence and low-virulence *M hyopneumoniae* strains (Villarreal and others 2011), less is known about virulence and genetic diversity of *M hyopneumoniae* strains used to manufacture bacterins and their relationship with *M hyopneumoniae* field strains. Villarreal and others (2012) mentioned that most of commercial vaccines are based on J strain; however, in a preliminary genetic characterisation of *M hyopneumoniae* bacterins, the authors have found other genetic subtypes of *M hyopneumoniae* in commercial vaccines available in the authors’ country (Pereyra and others 2012).

*M hyopneumoniae* genetic diversity has been reported around the world by using different molecular techniques (Stakenborg and others 2005, 2006; de Castro and others 2006; Tamiozzo and others 2011). From all typing techniques, Multiple-Locus Variable number tandem repeat Analysis (MLVA) seems to be a suitable molecular tool to detect *M hyopneumoniae* genetic subtypes (de Castro and others 2006, Vranckx and others 2011) since the isolation of the agent is not required as each locus is specifically amplified by PCR.

Due to the lack of information about both the genetic characteristics of commercial vaccines and their relationship with *M hyopneumoniae* field strains, the objective of this study was to identify genetic subtypes of some *M hyopneumoniae* bacterins and to compare them with *M hyopneumoniae* field strains present in the authors’ country.

## Materials and methods

This work was performed at the Laboratory of Animal Pathology of the Faculty of Agronomy and Veterinary Sciences (UNRC, Río Cuarto, Córdoba, Argentina), according to the international guidelines of the Council for International Organizations of Medical Sciences (CIOMS).

### Bacterins and bronchoalveolar lavage DNA

Six *M hyopneumoniae* bacterins that are commercialised in Argentina (and also in other countries) were analysed. In order to know the *M hyopneumoniae* strains used for the bacterins, information was retrieved from vaccine manufacturer companies. Bacterins A and D corresponded to strain J, but for bacterins B, C, E and F, no information was available. DNA from the *M hyopneumoniae* bacterins was extracted with a commercial kit (QIAamp Stool Mini Kit, Qiagen) to avoid the interference of possible PCR inhibitors.

In order to compare the allelic profiles of *M hyopneumoniae* bacterins with *M hyopneumoniae* field strains, DNA from 28 bronchoalveolar lavages (BAL) positive for the pathogen were included in the analysis. BAL corresponded to 22-week-old pigs at slaughter from three herds from centre-south of Córdoba province, and DNA was extracted using a commercial kit (DNAzol, Invitrogen) according to the manufacturer’s instructions. Before MLVA, DNA from *M hyopneumoniae* bacterins and field strains were tested by a species-specific nested PCR (nPCR; Calsamiglia and others 1999) to check that *M hyopneumoniae* DNA was present and able to be amplified by PCR. All the samples rendered positive results.

### Multiple-Locus Variable number tandem repeat Analysis

The MLVA scheme for the regions *p146R1*, *H4*, *H5* and *p95* was performed according to de Castro and others (2006). The amplicons were resolved in 2 per cent agarose gel run at 150 V for 3.5 hours and stained with SYBR Green I (Invitrogen).

Regarding the region *p146R3*, it was analysed by the nPCR developed by Tamiozzo and others (2011) for the first round of amplification, and the primers and conditions described by Mayor and others (2007) for the second round. The amplicons were purified (Puriprep-GP Kit, Inbio Highway, Tandil, Argentina), quantified (NanoDrop ND-1000, Thermo Fisher Scientific, Wilmington, Delaware, USA) and sequenced (ABI 3130*xl*; Applied Biosystems, Foster City, California, USA) with the primers used in the second round (Mayor and others 2007). The number of serine repeats (encoded by the codons TCT, TCA and TCC) was determined by viewing *p146R3* sequences with the BioEdit software (Hall 2007).

## Results

**FIG 1:**
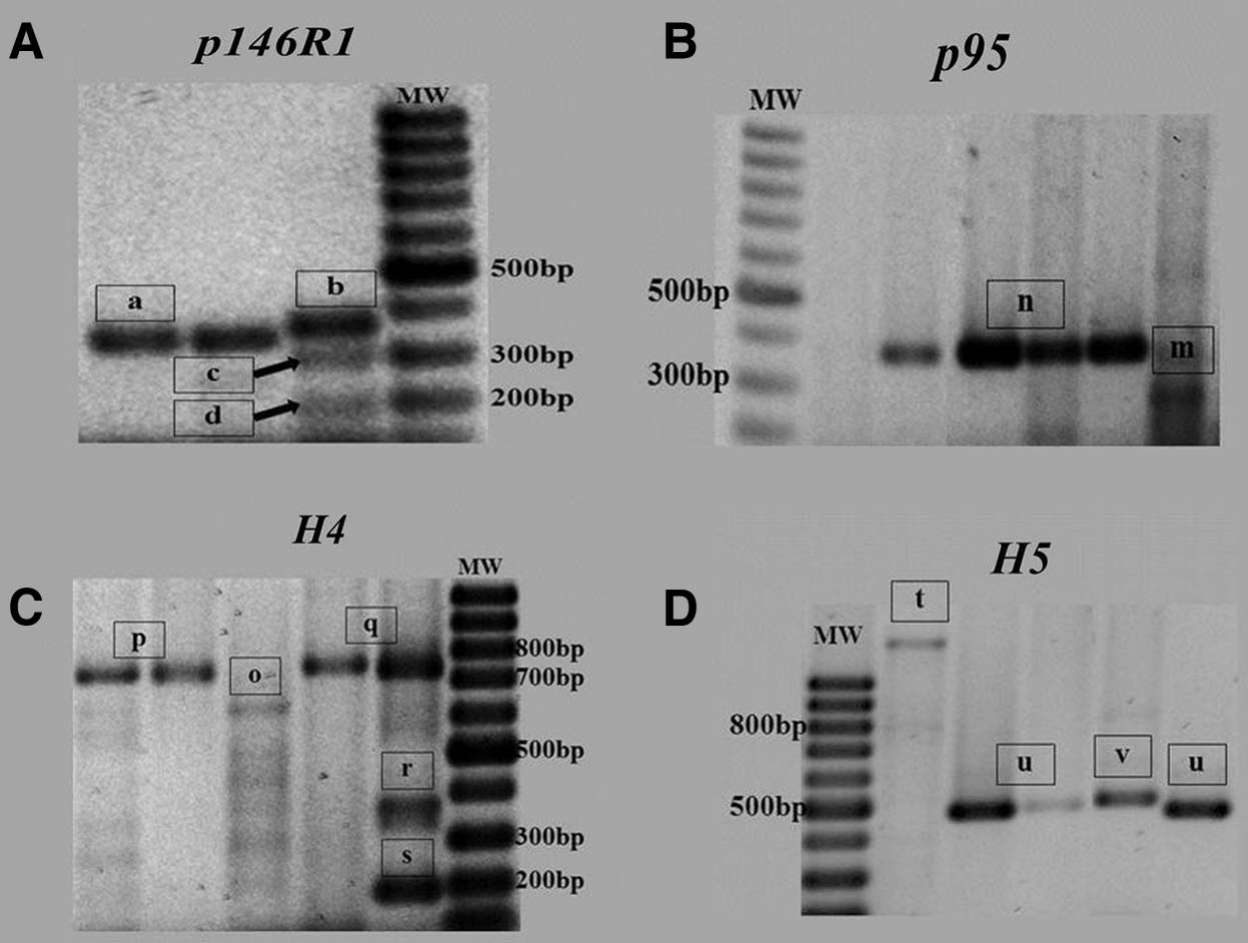
PCR results for loci *p146R1* (A), *p95* (B), *H4* (C) and *H5* (D). Numbers inside the boxes identify the alleles. MW, DNA ladder with fragments ranging from 100 bp to 1000 bp

Different *M hyopneumoniae* genetic subtypes were identified in the analysed samples. All the loci analysed rendered positive results with DNA samples obtained from *M hyopneumoniae* bacterins. In these loci, some alleles were identified among both bacterin and clinical samples (Fig 1), but others were found only within a particular kind of samples (Table 1).

**TABLE 1:** Alleles identified in each locus in the different samples analysed

	Herd A							Herd B									Herd C												Bacterins					
Loci	1	2	3	4	5	6	7	8	9	10	11	12	13	14	15	16	17	18	19	20	21	22	23	24	25	26	27	28	A	B	C	D	E	F
*P146R1*	–	a	–	a	–	a	–	–	a	a	–	a	–	a	a	a	–	–	–	–	–	a	–	a	–	a	a	–	a	b,c,d	a	–	–	–
*P146R3* (nested)	g	k	k	k	k	k	k	h	h	h	g	g	g	e	f	g	h	g	h	h	j	i	h	h	i	i	h	h	j	l	l	j	i	l
*P95*	–	m	–	m	–	m	–	–	–	m	m	m	m,n	–	m	m	m,n	–	–	–	–	m	–	m	–	–	–	–	–	m	–	–	m	m
*H4*	–	o	–	o	–	o	–	o	o	p	p	p	–	–	–	p	–	–	o	–	–	q	–	p	–	–	o	–	–	q,r,s	–	–	–	–
*H5*	t	u	–	–	–	u	–	–	–	v	–	v	t,v,w	u	u	u	–	t,v	–	–	–	v	–	v	u	u	u	–	–	u	–	–	–	u

Amplicons sizes (approximately) of probable alleles according to the loci studied: ***p146R1***: allele a (350 bp); allele b (370 bp); allele c (300 bp); allele d (200 bp). ***p146R3***: allele e (10 serines repeat); allele f (12 serines repeat); allele g (14 serines repeat); allele h (16 serines repeat); allele i (17 serines repeat); allele j (18 serines repeat); allele k (19 serines repeat); allele l (21 serines repeat). ***p95***: allele m (280 bp); allele n (350 bp). ***H4***: allele o(640 bp); allele p (700 bp); allele q (750 bp); allele r (370 bp); allele s (190 bp). ***H5***: allele t (more than 1000 bp); allele u (500 bp); allele v (520 bp); allele w (800 bp)

In a few loci were observed more than one allele in the same sample, in the case of a bacterin (locus *p146R1* in bacterin B) and four BAL samples (loci *p95* and *H5* in sample 13; locus *p95* in sample 17; locus *H5* in sample 18).

Many loci could not be amplified in some samples, but *p146R3*, which was the one analysed in an nPCR format, was the only locus able to be amplified in all samples. Therefore, the analysis of that polyserine repeat was feasible in all samples, and revealed that locus *p146R3* was the most polymorphic (Table 1).

## Discussion

Different *M hyopneumoniae* genetic subtypes, among and within herds and also among bacterin strains, were detected using MLVA. Despite the fact that the characterisation could not be performed for all loci, the results obtained contribute to the knowledge of the genetic diversity of the vaccines most used for pig immunisation against *M hyopneumoniae*, of which, there have been few previous characterisations (Lin 2001, de Castro and others 2006). In the MLVA scheme used in the present study, the region that showed more polymorphism was *p146R3*, followed by *H4*, *H5*, *p146R1* and *p95*. These results are in agreement with those of de Castro and others (2006) who selected four of these regions to unambiguously discriminate J, 7448, 7422 and PMS *M hyopneumoniae* strains.

Most of the loci could not be amplified in all samples. This could be due to variability in the primer-binding sites or, most probably, to the lower sensitivity of the PCRs used, since the nPCR used for *M hyopneumoniae* detection (Calsamiglia and others 1999) and the other for the analysis of *p146R3* locus rendered positive results with all of the 28 DNA samples.

Although de Castro and others (2006) suggested that developed PCR assays could have sufficient sensitivity for *M hyopneumoniae* typing from clinical samples, a complete characterisation by MLVA is not always possible using conventional PCR, even when working with BAL samples. Kuhnert and others (2011) noticed that successful genotyping was dependent on a sufficiently high concentration of *M hyopneumoniae* DNA in lung samples from wild boar. In this regard, the authors have recently reported the need to increase the sensitivity of some of the PCRs used for MLVA typing of this pathogen (Tamiozzo and others 2013). Sensitivity could be increased also using touchdown PCR (Korbie and Mattick 2008) and/or capillary electrophoresis (Vranckx and others 2011). This would allow *M hyopneumoniae* typing from minimally invasive samples (such as nasal swabs) without killing animals or performing invasive sampling (Tamiozzo and others 2013).

Charlebois and others (2014) reported that they were unable to type *M hyopneumoniae* vaccine strains using PCR-restriction fragment length polymorphism (RFLP) due to the absence of amplification of the *p146* gene. In the present paper, the use of the nPCR format for the *p146R3* locus was shown to be advantageous and allowed the typing from all samples.

Interestingly, in some samples, more than one allele seemed to be present in the same locus. This fact could be due to unspecific primer annealing or to the presence of different *M hyopneumoniae* genotypes in the same sample. An unspecific annealing of the primers is considered highly unlikely. The presence of different genotypes of *M hyopneumoniae* has been reported previously. The authors found two probable alleles present in one pig BAL sample when analysing the locus *H4* from *M hyopneumoniae* in a previous study in the authors’ country (Tamiozzo and others 2011). Furthermore, Vranckx and others (2011) demonstrated simultaneous infection with multiple *M hyopneumoniae* strains both by the presence of double alleles in the electropherograms of the MLVA loci of clinical samples and by the presence of different strains isolated from the same animals.

According to manufacturer’s information, bacterins A and D are based on *M hyopneumoniae* strain J, and the present results are in accordance to that, because *p146R3* region showed a repeat motif with 18 serines, agreeing with previous reports (de Castro and others 2006, Mayor and others 2007) and the available data for that strain in National Center for Biotechnology Information (NCBI) database. For bacterin A, the results for *p146R1* also corresponded to strain J, but, unfortunately, the other variable number tandem repeats could not be analysed due to the presence of null alleles.

Among *M hyopneumoniae* field strains present in the BAL samples, the *p146R3* allele with the 18 serine repeat motif was not identified. Although this allele has not been previously found either in *M hyopneumoniae* field strains from Argentina (Tamiozzo and others 2011) or in *M hyopneumoniae* vaccine strains from Brazil (de Castro and others 2006), it has been detected in *M hyopneumoniae* field strains from Europe (Mayor and others 2007, Savic and others 2010). Furthermore, the *M hyopneumoniae* non-pathogenic strain J was isolated from a pig herd in the UK in 1963 (Villarreal and others 2012), and maybe, this genotype is not present in South America. However, more studies have to be performed to elucidate this.

Regarding bacterins B, C and F, they all showed a 21-serine repeat motif in the *p146R3* analysis. The same number of serine repeats has been reported in the *M hyopneumoniae* strain 232 isolated in the USA and in field strains from Europe and Argentina (Minion and others 2004, Mayor and others 2007, Savic and others 2010, Tamiozzo and others 2011, 2013), but not in strains isolated in Brazil (Vasconcelos and others 2005) that were analysed by de Castro and others (2006). Additionally, bacterins B and F showed the same alleles in *p95* and *H5* regions (500 bp), but bacterin B seems to be made from more than one strain, since three possible alleles were found in each of the two loci (*p146R1* and *H4*). Bacterins B and C could be distinguished with regard to the alleles present at locus *p146R1*.

Although a recent study has pointed out that a locus (which encodes a hypothetical protein) could be associated with *M hyopneumoniae* virulence (Charlebois and others 2014), at present, a molecular marker able to identify high-virulence and low-virulence *M hyopneumoniae* strains does not exist, and therefore, differences in performance of vaccination in each particular herd cannot be explained that way.

In the present paper, the utility of MLVA for *M hyopneumoniae* typing in clinical samples and bacterins was demonstrated, particularly when a high-sensitivity method such as nPCR is used. Furthermore, the results obtained showed the presence of more than one *M hyopneumoniae* genotype in some pigs and also in one of the bacterins analysed. It is also worth noting that MLVA typing allowed the distinction among circulating field strains and also when comparing them with vaccine strains, which, knowing the relatedness among them, could be useful in the research of the efficacy of the vaccines.
